# Recurrent spontaneous clearance of psoriasis during exacerbations of concomitant chronic spontaneous urticaria

**DOI:** 10.1002/ccr3.2930

**Published:** 2020-05-25

**Authors:** Eli Magen, Tinatin Chikovani

**Affiliations:** ^1^ Allergy and Clinical Immunology Unit Medicine C Department Barzilai Medical Center Ben Gurion University of Negev Ashkelon Israel; ^2^ Department of Immunology Tbilisi State Medical University Tbilisi Georgia

**Keywords:** chronic, psoriasis, remission, spontaneous, urticaria

## Abstract

In this case report, we describe a patient with a chronological relationship between exacerbations of chronic spontaneous urticaria and remissions of concomitant generalized plaque psoriasis.

## Graphical abstract

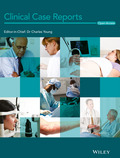

## 1 INTRODUCTION

Little is known about the potential links between psoriasis and chronic spontaneous urticaria (CSU).^1^, ^2^ Here, we describe a patient with long‐standing plaque psoriasis, who repeatedly experienced an almost complete but transient spontaneous clearance of psoriasis after exacerbations of concomitant CSU.

## 2 CASE REPORT

A 36‐year‐old female patient had a 24‐year history of moderate, generalized plaque psoriasis covering 10% of her body surface area. She had been heavily treated in the past for psoriasis with multiple medications, including tacalcitol, corticosteroids, cyclosporine, and UVB phototherapy, which yielded only a partial response.

In August 2016, she presented to our allergy/immunology clinic for daily generalized hives during the past 2 months. On examination, the patient had unremarkable vital signs, and the Psoriasis Area and Severity Index (PASI) score was 9.

She denied any allergic diseases, as well as urticaria or angioedema attacks in the past. Family history, medical history, and prescribed drugs were unremarkable. In the laboratory examinations, whole blood count, liver function tests, renal function tests, serum electrolytes, erythrocyte sedimentation rate, CRP, thyroid function tests, and C3 and C4 were normal; the antinuclear antibodies were negative, and total IgE was 95.7 kU/L. The urticaria activity score (UAS) was 4, and the 7‐day UAS (UAS7) was 36.

The patient was diagnosed with CSU, and daily fexofenadine (180 mg) was initiated with a good clinical response. After 6 months, she discontinued the antihistamine medication.

In September 2016, during the next exacerbation of CSU, the patient had noticed spontaneous remission of her psoriatic eruption. In December 2016, her psoriasis had almost completely cleared.

In July 2017, she returned the dermatology clinic due to the reappearance of psoriatic lesions. The affected body surface area was approximately 10% (PASI score of 9). Ustekinumab was prescribed, but the patient did not pick the medication up from the pharmacy, and instead applied topical tacalcitol and corticosteroids. Since May 2017 and until February 2019, the patient experienced a complete remission of CSU.

In March 2019, the patient visited our allergy and immunology clinic, reporting that the urticarial lesions and lip angioedema had relapsed 2 months earlier. The patients' recorded weekly UAS7 score was 32. She was taking 180 mg/d fexofenadine, without symptom relief. She also reported that 3 weeks after the appearance of urticarial lesions, her psoriasis had significantly improved.

The patient continued fexofenadine (180 mg b.i.d.) with a satisfactory clinical response. In August 2019, the patient returned to our clinic for follow‐up; she was almost completely cleared from psoriatic lesions (absolute PASI < 2; PASI 90 response).

The chronological sequences of the two disease conditions are presented in Table 1.

**TABLE 1:** The chronological sequences of chronic spontaneous urticaria and generalized plaque psoriasis

	CSU	UAS	UAS7	Psoriasis	PACI	CSU treatment	Psoriasis treatment
10.1992‐06.2016	No	0	0	Generalized plaque psoriasis	5‐11		Tacalcitol, topical corticosteroids, cyclosporine, UVB phototherapy
17.08.2016	CSU diagnosed	4		Generalized plaque psoriasis	9	Fexofenadine 180 mg qd	Topical corticosteroids
05.09.2016	CSU exacerbation		36	Generalized plaque psoriasis	5	Fexofenadine 180 mg qd	None
08.12.2016	CSU exacerbation	5	34	Spontaneous remission	<2	Fexofenadine 180 mg bid	None
11.03.2017	CSU clinical improvement	2	14		<2	Fexofenadine 180 mg qd, 25.03.2017 discontinued	None
05.2017‐02.2019	CSU complete remission		0			None	
07.2017	CSU complete remission	0	0	Reappearance of generalized plaque psoriasis	9		Topical corticosteroids
04.03.2019	CSU exacerbation	5				Fexofenadine 180 mg bid	None
19.03.2019	CSU active		34		4	Fexofenadine 180 mg bid	None
12.08.2019	CSU active	3		Almost completely cleared from psoriatic lesions	<2	Fexofenadine 180 mg qd	None

Abbreviations: CSU, chronic spontaneous urticarial; PASI, Psoriasis Area and Severity Index; UAS, urticaria activity score; UAS7, the 7 d urticaria activity score.

## 3 DISCUSSION

Here, we report a chronological relationship between exacerbations of CSU and remissions of concomitant generalized plaque psoriasis. Although a chronological relationship cannot be interpreted as causal, this possibility cannot be ruled out. We found no reports on the correlation between disease severity during CSU and psoriasis concomitance in the existing medical literature.

Mast cell (MC) activation is one of the earliest morphological changes in developing both psoriatic lesions^3^ and CSU.^4^ Moreover, induction of the Koebner reaction in healthy‐looking skin of patients with psoriasis is associated with an increase in MC numbers, which peaks with the appearance of psoriatic plaques.^5^ Compared to CSU, psoriasis is considered to be a Th1‐driven disease characterized by the infiltration of several T‐cell subsets, dendritic cells, neutrophils, natural killer T cells, and MCs.^6^ Although MCs have been thought to be proinflammatory in models of psoriasis, this is not always the case. They can occasionally play the role of immune suppressors/modulators in several diseases.^7^ Human MCs have been shown to express and release the inhibitory cytokines IL‐10,^8^ TGF‐β,^9^ and IL‐37.^10^ MCs have direct effects on T cells, such as the induction of T regulatory cells, which suppress inflammation.^11^ MC proteases, such as tryptase and chymase, degrade proinflammatory cytokines and alarmins, which results in an immunoregulatory effect.^12^

It was previously reported that an inhibitor of Hedgehog (Hh) signaling plays a role in epidermal hyperproliferation in psoriasis^13^ and that the neurofibromatosis type 1 (NF1) tumor suppressor gene expression is poor in keratinocytes of psoriatic lesions.^14^ Ketotifen was shown to be effective in successfully blocking NF1‐associated neurofibromas by blocking mast cell degranulation.^15^ A similar mechanism might have contributed to the suppression of psoriatic epidermal hyperproliferation in our patient during antihistamine treatment of CSU exacerbations.

The observed association between psoriasis remission and CSU exacerbation has no biologically plausible link and might be casual.

Nonetheless, if a case represents a real negative connection between the two diseases, CSU would be found at a lower frequency in patients with psoriasis and vice versa. This link should now be investigated using large epidemiological surveys.

## CONFLICT OF INTEREST

None declared.

## AUTHOR CONTRIBUTION

Eli Magen: analyzed and interpreted the patient's data regarding the chronological sequences of chronic spontaneous urticaria and concomitant generalized plaque psoriasis. Eli Magen and Tinatin Chikovani: discussed the clinical case and contributed to the final manuscript. Both authors: read and approved the final manuscript.

## References

[1] Caldarola G , De Simone C , Talamonti M , et al. Prevalence of cutaneous comorbidities in psoriatic patients and their impact on quality of life. Eur J Dermatol. 2019;29:192‐196. 30973328 10.1684/ejd.2019.3529

[2] Magen E , Mishal J , Schlesinger M . Clinical and laboratory features of chronic idiopathic urticaria in the elderly. Int J Dermatol. 2013;52:1387‐1391. 23834467 10.1111/ijd.12109

[3] Harvima IT , Nilsson G , Suttle M‐M , Naukkarinen A . Is there a role for mast cells in psoriasis? Arch Dermatol Res. 2008;300:461‐478. 18719932 10.1007/s00403-008-0874-x

[4] Bracken SJ , Abraham S , MacLeod AS . Autoimmune theories of chronic spontaneous urticaria. Front Immunol. 2019;10:627. 30984191 10.3389/fimmu.2019.00627 PMC6450064

[5] Toyry S , Fraki J , Tammi R . Mast cell density in psoriatic skin. The effect of PUVA and corticosteroid therapy. Arch Dermatol Res. 1988;280:282‐285. 3178285 10.1007/BF00440601

[6] Gaspari AA . Innate and adaptive immunity and the pathophysiology of psoriasis. J Am Acad Dermatol. 2006;54(3 Suppl 2):S67‐S80. 16488332 10.1016/j.jaad.2005.10.057

[7] Mekori YA , Hershko AY . T cell‐mediated modulation of mast cell function: heterotypic adhesion‐induced stimulatory or inhibitory effects. Front Immunol. 2012;3:6. 22566892 10.3389/fimmu.2012.00006 PMC3342371

[8] Ishizuka T , Okayama Y , Kobayashi H , Mori M . Interleukin 10 is localized to and released by human lung mast cells. Clin Exp Allergy. 1999;29:1424‐1432. 10520066 10.1046/j.1365-2222.1999.00636.x

[9] Kanbe N , Kurosawa M , Nagata H , Saitoh H , Miyachi Y . Cord blood‐derived human cultured mast cells produce transforming growth factor β1. Clin Exp Allergy. 1999;29:105‐113. 10.1046/j.1365-2222.1999.00459.x 10051709

[10] van de Veerdonk FL , Netea MG . New insights in the immunobiology of IL‐1 family members. Front Immunol. 2013;4:167. 23847614 10.3389/fimmu.2013.00167 PMC3703542

[11] Lu LF , Lind EF , Gondek DC , et al. Mast cells are essential intermediaries in regulatory T‐cell tolerance. Nature. 2006;442:997‐1002. 16921386 10.1038/nature05010

[12] Piliponsky AM , Chen CC , Rios EJ , et al. The chymase mouse mast cell protease 4 degrades TNF, limits inflammation, and promotes survival in a model of sepsis. Am J Pathol. 2012;181:875‐886. 22901752 10.1016/j.ajpath.2012.05.013 PMC3432424

[13] Tas S , Avci O . Rapid clearance of psoriatic skin lesions induced by topical cyclopamine. A preliminary proof of concept study. Dermatology. 2004;209:126‐131. 15316166 10.1159/000079596

[14] Karvonen SL , Koivunen J , Nissinen M , Ylä‐Outinen H , Björkstrand AS , Peltonen J . Neurofibromatosis type 1 tumour suppressor gene expression is deficient in psoriatic skin in vivo and in vitro: a potential link to increased Ras activity. Br J Dermatol. 2004;150:211‐219. 14996090 10.1111/j.1365-2133.2004.05767.x

[15] Riccardi VM . Ketotifen suppression of NF1 neurofibroma growth over 30 years. Am J Med Genet A. 2015;167:1570‐1577. 25974154 10.1002/ajmg.a.37045

